# Serosurveillance for Livestock Pathogens in Free-Ranging Mule Deer (*Odocoileus hemionus*)

**DOI:** 10.1371/journal.pone.0050600

**Published:** 2012-11-27

**Authors:** Annette Roug, Pamela Swift, Steven Torres, Karen Jones, Christine K. Johnson

**Affiliations:** 1 Wildlife Health Center, School of Veterinary Medicine, University of California Davis, Davis, California, United States of America; 2 California Department of Fish and Game, Rancho Cordova, California, United States of America; University of Georgia, United States of America

## Abstract

Routine disease surveillance has been conducted for decades in mule deer (*Odocoileus hemionus*) in California for pathogens shared between wildlife and domestic ruminants that may have implications for the animal production industry and wildlife health. Deer sampled from 1990 to 2007 (n = 2,619) were tested for exposure to six pathogens: bluetongue virus (BTV), epizootic hemorrhagic disease virus (EHDV), bovine viral diarrhea virus (BVDV), *Leptospira* spp., *Anaplasma* spp. and *Brucella* spp. We evaluated the relationship between exposure to these pathogens and demographic risk factors to identify broad patterns in seroprevalence across a large temporal and spatial scale. The overall seroprevalence for the entire study period was 13.4% for BTV, 16.8% for EHDV, 17.1% for BVDV, 6.5% for *Leptospira* spp., 0.2% for *Brucella* spp., and 17% for *Anaplasma* spp. Antibodies against BTV and EHDV were most prevalent in the deer populations of southern California. Antibodies against *Leptospira* spp. and *Anaplasma* spp. were most prevalent in coastal and central northern California whereas antibodies against BVDV were most prevalent in central-eastern and northeastern California. The overall seroprevalence for *Anaplasma* spp. was slightly lower than detected in previous studies. North and central eastern California contains large tracts of federal land grazed by livestock; therefore, possible contact between deer and livestock could explain the high BVDV seroprevalence found in these areas. Findings from this study will help to establish baseline values for future comparisons of pathogen exposure in deer, inform on long-term trends in deer population health and provide relevant information on the distribution of diseases that are shared between wildlife and livestock.

## Introduction

Routine disease surveillance in mule deer (*Odocoileus hemionus*) has been conducted in California for decades. Surveillance has targeted pathogens that can be shared between wildlife and domestic ruminants, such as bluetongue viruses (BTV), epizootic hemorrhagic disease viruses (EHDV), bovine viral diarrhea viruses (BVDV), *Leptospira* spp., *Brucella* spp. and *Anaplasma* spp. Diseases caused by these pathogens not only have implications for the animal production industry in California, but also are relevant to the health of wild ungulates and can inform management of deer hunting activities and conservation.

Bluetongue viruses and EHDV are related orbiviruses that are transmitted by *Culicoides* spp. vectors. In the United States, bluetongue is primarily a disease of sheep [1] and clinical infection in cattle is rare [2]. Bluetongue virus serotypes 10, 11, 13 and 17 have previously been identified on dairy farms in the northern Central Valley of California [3]. Mule deer are susceptible to infection with BTV but experimental infections in black-tailed deer (*Odocoileus hemionus columbianus*) failed to produce clinical signs [4]. Recent emergence of pathogenic bluetongue serotypes in Europe may be cause for concern [5], and some of these serotypes can also infect wild ruminants [6]–[8]. The risk for clinical EHD in livestock is generally considered low although several outbreaks of clinical EHDV have occurred in cattle in recent years [9]–[11]. Serotypes EHDV-1 and EHDV-2 have been isolated from calves in San Joaquin Valley of California [12]. Outbreaks of EHDV with high mortality rates have been reported in white-tailed deer [13]–[15] and the apparent cyclical occurrence of outbreaks in wild deer are thought to be caused by variations in herd immunity and vector populations [16]. Serotype EHDV-2 specifically has been attributed to mortalities in two mule deer and one white-tailed deer (*Odocoileus virginianus*) in neighboring Arizona [17]. Recently, a reassortment EHD virus, the EHDV-6, strain Indiana, containing segments of both North American EHDV-2 (Alberta strain) as well as exotic EHDV-6 (CSIRO 753 strain) was detected in white-tailed deer in Indiana, Illinois, Missouri, Texas, Kansas, Michigan and Arkansas [18], [19]. Although this novel virus has not been isolated from livestock, this finding together with the emergence of pathogenic BTV strains in Europe and recent outbreaks of clinical EHD in cattle highlights the importance of disease monitoring in livestock and wild deer populations.

Bovine viral diarrhea virus (BVDV) is predominantly a pathogen of cattle although deer are susceptible to infection [20]. The main concern for BVD in cattle are persistently infected calves that continuously shed virus [21]. Experimental studies showed that deer also are capable of producing persistently infected offspring [22], [23]. Although there is no current U.S. government eradication program [24], researchers have raised the concern that wildlife BVD could be an impediment for disease control in cattle [25], [26].

The spirochete *Leptospira* spp. affects a range of mammalian hosts, including wildlife and domestic animals. *Leptospira* spp. are shed in urine and can survive for months in moist environments [27], [28]. Unvaccinated cattle are susceptible to leptospirosis and clinical signs can be severe [29]. Disease due to leptospirosis is rare in free-ranging deer [27] but has been reported in farmed-deer in New Zealand [30]. *Brucella* spp. is another bacterial zoonosis, which can similarly infect a range of wild and domestic animal species. Brucellosis has been officially eradicated from California since 1997 [31] but is monitored in wildlife because of its importance to the livestock industry.

*Anaplasma* spp. are obligate intra-erythrocytic, arthropod-borne bacteria that occur in tropical, subtropical and some temporal regions [32]. The predominant vector for livestock anaplasmosis in the U.S. are *Dermacentor* spp. ticks, and *Anaplasma marginale* can cause fatal disease in adult cattle [33]. There is concern that the geographical distribution of arthropod vectors may be shifting with climate change [34]. In 2000, anaplasmosis was confirmed in Saskatchewan bison, a location far outside the expected range [35]. In California, exposure or infection with *A. marginale* [36], *A. ovis* [37], [38] and *A. phagocytophilum* has been confirmed in deer [38]. Black-tailed deer are considered more susceptible to infection than mule deer [39] although both can develop ricksettsemia of several weeks duration [40].

Surveys of exposure to BTV, EHDV, *Leptospira* spp., *Anaplasma* spp. and *Brucella* spp. in California deer have not included new data for nearly two decades [36], [41], [42] and very limited information is available on the seroprevalence of BVDV in California deer [43]. The goals of this retrospective study were to 1) estimate baseline seroprevalence and distribution of BTV, EHDV, BVDV, *Leptospira* spp., *Anaplasma* spp., and *Brucella* spp. in California deer from 1990 to 2007, 2) evaluate demographic factors that may be associated with exposure to these pathogens, and 3) identify high risk areas where disease exposure in deer is prevalent.

## Methods

**Figure 1:**
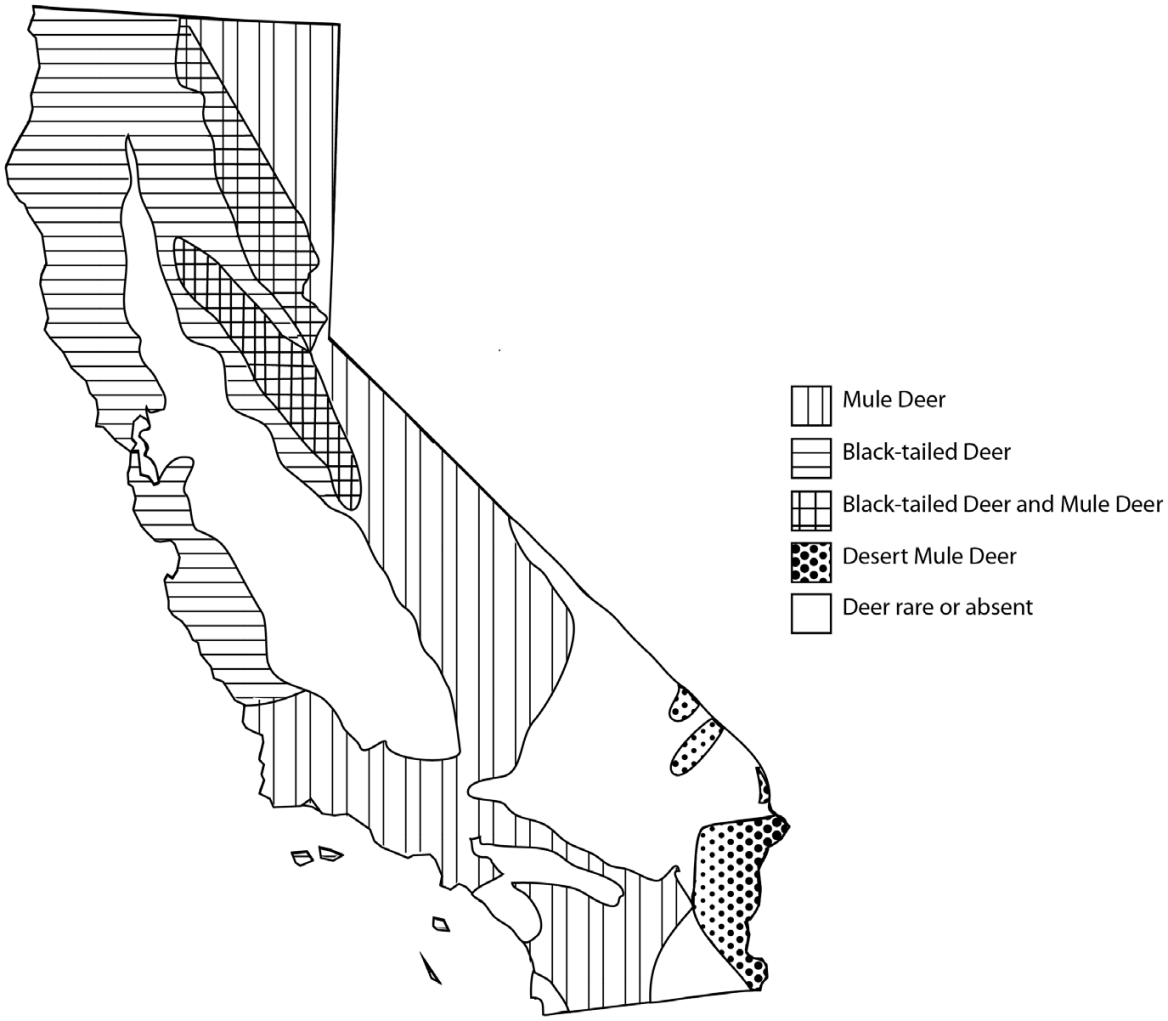
Deer subspecies. Distribution of deer and estimated ranges for specific deer subspecies in California. Black-tailed deer (*Odocoileus hemionus columbianus*) are considered genetically distinct. For the purpose of this analysis, the 4 mule deer subspecies; California mule deer (*Odocoileus hemionus californicus*), Inyo mule deer (*Odocoileus hemionus inyoensis*), Southern mule deer (*Odocoileus hemionus fuliginatus*) and Rocky Mountain mule deer (*Odocoileus hemionus hemionus*) were grouped into one “Mule deer” category. Desert mule deer (*Odocoileus hemionus eremicus*) were analyzed separately due to their distinct ecology. Adapted from: California Department of Fish and Game (1999): A Sportsman’s guide to improving deer habitat in California (http://www.dfg.ca.gov/wildlife/hunting/deer/sportsmanguide.html).

Disease surveillance activities were conducted by the California Department of Fish and Game (CDFG) in collaboration with the Department of Food and Agriculture (CDFA) between 1990 and 2007 across 44 counties in California. All capture and animal handling was overseen and approved by the CDFG. Serum samples were collected from 2,619 deer, including 943 black-tailed deer (*Odocoileus hemionus columbianus*) and 5 mule deer subspecies; California mule deer (*Odocoileus hemionus californicus*, n = 135), Inyo mule deer (*Odocoileus hemionus inyoensis*, n = 220), Southern mule deer (*Odocoileus hemionus fuliginatus*, n = 329), Rocky Mountain mule deer (*Odocoileus hemionus hemionus*, n = 936) and Desert mule deer (*Odocoileus hemionus eremicus*, n = 56), which are known locally as Burro deer. Genetic analysis indicates that black-tailed deer are a distinct subspecies whereas the mule deer ecotypes are a result of more recent geographic isolation [44]. Some taxonomic confusion persists with respect to mule deer subspecies and their common names so, for the purposes of this analysis, California mule deer, Inyo mule deer, Southern mule deer and Rocky Mountain mule deer were grouped together as “mule deer”. Desert mule deer were categorized separately from other mule deer because of their distinct ecology and geographic isolation within area 8 in California. The estimated ranges for the different deer subspecies in California using this classification are shown in Fig. 1. Most samples (n = 2,564) were collected from apparently healthy live free-ranging deer during capture events, whereas 55 blood samples were obtained from dead deer necropsied at the California Department of Fish and Game’s Wildlife Investigation Laboratory in Rancho Cordova, California. Necropsy results were available for 40/55 deer and causes of death included pneumonia, parasitism and trauma. Two deer had lesions indicative of hemorrhagic disease but no final diagnoses were confirmed.

**Figure 2:**
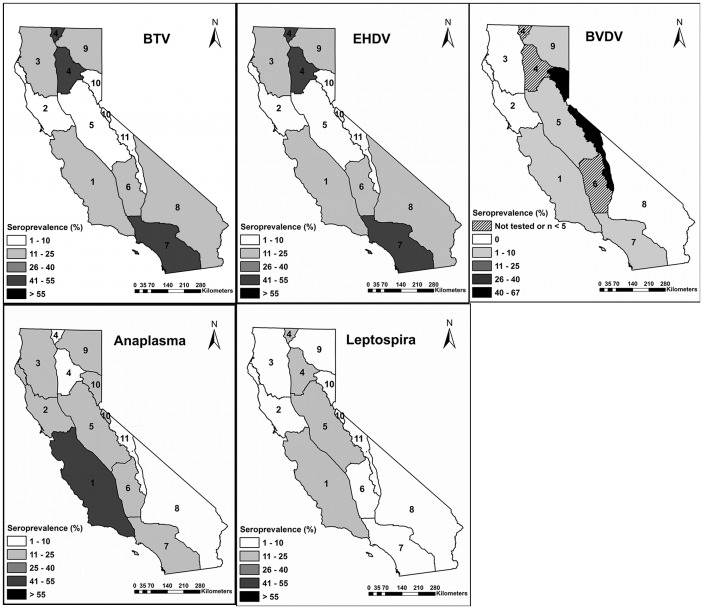
Seroprevalences by deer assessment unit (area). Ranges of average seroprevalences for bluetongue viruses (BTV), epizootic hemorrhagic disease viruses (EHDV), bovine viral diarrhea virus (BVDV), *Leptospira* spp. and *Anaplasma* spp. among deer sampled between 1990 and 2007 in 11 deer assessment units (areas) in California.

Methods for live captures included net-gunning from a helicopter (Coda Netgun, Coda Enterprises, Mesa, AZ), driving deer into a line net on the ground, clover traps, and free-range darting (Pneu-dart, Williamsport, PA) using a combination of 2.2 mg/kg tiletamine and zolazepam (Telazol®, Fort Dodge Animal Health) and 2.2 mg/kg xylazine (generic) [45]. Immobilized deer were hobbled and blindfolded, and blood samples were collected by jugular venipuncture and placed directly into serum separator tubes. Blood was allowed to clot at room temperature for 2–4 hours; then centrifuged at 3,000 rpm for ten minutes. Serum was removed, placed into plastic vials, and refrigerated or frozen at −15 C until transport to the laboratory. Excess serum was archived at −80 C. Blood from dead deer was obtained from the heart, centrifuged at 3,000 rpm for ten minutes, and processed as noted above. Data on age class (>2 or <2 years), sex, deer subspecies, year and location (county) were also recorded. The deer population in California is managed according to 11 deer assessment units, which are areas with distinct habitat and migratory behavior of deer, and deer were therefore categorized by these 11 management “areas” (Fig. 2).

Samples were submitted to the California Animal Health and Food Safety Laboratory (CAHFS, University of California, School of Veterinary Medicine, Davis, California). Samples were tested for antibodies against BTV using the agar gel immunodiffusion test (AGID, Veterinary Medical Research and Development, Inc., Pullmann WA) for the 845 samples collected prior to 1994 and with the competitive enzyme-linked immunosorbent assay test (cELISA, Veterinary Medical Research and Development, Inc., Pullmann WA) for the 1,646 samples obtained after 1994. The samples were classified as positive or negative according to the manufacturer’s instructions [46], [47]. Both tests have been widely used in domestic animals and wild ruminants, but cELISA is considered more accurate because of less cross reactivity with other orbiviruses such as EHDV [48], [49]. Samples were tested for EHDV (n = 2,507) by the agar gel immunodiffusion test and classified as positive or negative according to the manufacturer’s instructions (AGID, Veterinary Diagnostic Technology, Inc., Wheat Ridge, CO). This test has been shown to cross-react with BTV [4], [48], [50]. As part of a pilot study to investigate occurrence of pestiviruses in California wild deer, a subset of samples (n = 409) from 21 counties were tested for BVD-type 2 virus using the serum neutralization test as previously described [51]. Titers ≥16 on the BVD-type 2 serum neutralization test were considered positive. The test has not been validated in deer and cross-reactivity with BVD-type 1 [51] and the closely related Border disease virus [52] can occur.

A total of 2,349 samples were tested for *Leptospira interrogans serovars grippotyphosa*, *hardjo*, *pomona*, *icterohaemorrhagiae* and *canicola* with the microscopic agglutination test (MAT) [53], [54]. An antibody titer ≥100 was considered positive per established protocols for livestock [53] and other studies evaluating the leptospirosis seroprevalence in deer [55], [56]. The MAT is the most widely used serologic test for *Leptospira*, but there is significant cross-reactivity between serovars [53], especially at lower titers, and this test has not been validated in deer. Samples were tested for antibodies against *Brucella* spp. (n = 2,544) using the buffered acidified plate antigen (BAPA) test and reagents from USDA National Veterinary Services Laboratory, Ames, IA according to USDA Uniform Methods and Requirements [57]. As with the other diagnostic tests, the BAPA has not been validated in deer, and false positive results due to cross-reactivity with other pathogens, such as *Yersinia enterocolitica* O:9 have been observed [58], [59].

Sera were tested for antibodies to *Anaplasma marginale* (n = 2,403) using the complement fixation test (CF) for 88 samples [60] and the card agglutination test for 2,315 samples [61] with reagents from the USDA National Veterinary Services Laboratory. The CF test has been found to have varying sensitivity and specificity in cattle [62] and wild ruminants [63] and has largely been replaced by more accurate tests. The card test has high sensitivity in cattle [64] and black-tailed deer [63]. Although both tests target exposure to *Anaplasma marginale*, cross reactivity with other *Anaplasma* spp. occurs [65]. Recent studies using DNA sequencing have provided evidence that deer in the western U.S. can be infected with *A. ovis, A. phagocytophilum* and *A. marginale* [37], [38] so seropositive test results in our study population may indicate exposure to *Anaplasma marginale* or other *Anaplasma* species.

### Statistical Analysis

Descriptive statistics were used for preliminary comparisons of the prevalence of seropositive status for BTV, EHDV, BVDV, *Leptospira* spp., *Brucella* spp., and *Anaplasma* spp., by age, sex, deer subspecies (black-tailed deer, desert mule deer, other mule deer), deer assessment unit (area) and year of capture, and live versus dead status at time of sampling. A mixed effects logistic regression model was fitted for each pathogen independently with positive or negative serologic status as the outcome. The model was adjusted for sampling at varying intensities in different locations and years and dependence among samples caused by temporal trends in pathogen exposure was addressed by including robust standard errors clustered on capture year. Terms for the regression models were selected using forward selection, and individual predictors were retained or discarded using the Wald’s and likelihood ratio test [66]. Model fit was assessed using the Hosmer Lemeshow test [67]. The McNemar Chi-square test was used to statistically assess cross-reactivity between the serological tests for BTV and EHDV. The relationship between rainfall and the seroprevalences of EHDV and BTV was explored using gridded annual precipitation data from The Prism Climate Group (PRISM Climate Group, Oregon State University, http://prism.oregonstate.edu, accessed 2012 Sept 27). The average annual precipitation was calculated for each deer assessment unit (area) and year using the zonal statistics function in ArcMap (vs. 10, ESRI, Redlands, CA, USA) and correlated with the seroprevalence (no deer seropositive/no of deer tested) for the overall dataset and within each deer assessment unit for areas with ≥7 years sampled using the spearman rank correlation test. Only years in which at least 10 deer were tested within an area were included in these analyses. The statistical software intercooled STATA (vs. 10, StataCorp, College Station, TX, USA) was used for analyses, and ArcMap used to generate maps. For all statistical tests, *P*-values≤0.05 were considered significant.

## Results

The seroprevalence among all deer sampled over the entire study period was 13.4% for BTV, 16.8% for EHDV, 17.1% for BVDV, 6.5% for *Leptospira* spp., and 17.0% for *Anaplasma* spp. Antibodies against *Brucella* spp. were detected in four deer. The seroprevalence of BTV, EHDV, BVDV, *Leptospira* spp., *Anaplasma* spp., and *Brucella* spp. among age, sex, deer subspecies and regional categories (areas) are shown in Table 1.

**Table 1:** Unadjusted prevalence (%) by year, age, sex and subspecies of serologic positive status for BTV, EHDV, BVDV, *Leptospira* spp., *Anaplasma* spp., and *Brucella* spp. among deer sampled between 1990 and 2007 in 11 deer assessment units (areas) in California.

	BTV	EHDV	BVDV	*Leptospira* spp.	*Anaplasma* spp.	*Brucella* spp.
**Overall**	13.4 (334/2491)	16.8 (422/2507)	17.1 (70/409)	6.5 (153/2349)	17.0 (409/2403)	0.2 (4/2544)
**Age**						
<2	12 (56/460)	16 (72/459)	13 (8/64)	7 (31/427)	18 (77/432)	0.4 (2/466)
>2	15 (268/1830)	18 (325/1811)	15 (45/301)	6 (105/1688)	18 (306/1727)	0.1 (2/1840)
Unknown	5 (10/201)	11 (25/237)	39 (17/44)	7 (17/234)	11 (26/244)	0 (0/238)
**Sex**						
Male	19 (125/663)	24 (155/654)	8 (7/93)	10 (61/581)	23 (137/601)	0.2 (1/660)
Female	12 (207/1723)	15 (262/1720)	18 (52/294)	5 (83/1639)	15 (256/1674)	0.2 (3/1753)
Unknown	2 (2/105)	4 (5/133)	50 (11/22)	7 (9/129)	13 (16/128)	0 (0/131)
**Subspecies**						
Black-tailed deer	14 (124/870)	19 (166/859)	7 (8/115)	13 (104/773)	31 (253/805)	0.3 (3/890)
Mule deer	11 (166/1567)	13 (206/1594)	21 (62/294)	3 (49/1537)	10 (156/1550)	0.1 (1/1600)
Desert mule deer	81 (44/54)	93 (50/54)	Not tested	0 (0/39)	0 (0/48)	0 (0/54)
**Area**						
1	15 (77/497)	23 (114/489)	3 (2/61)	12 (53/435)	43 (199/461)	0.4 (2/503)
2	8 (6/80)	9 (7/80)	0 (0/8)	5 (3/65)	11 (8/76)	0 (0/79)
3	12 (8/65)	20 (13/65)	0 (0/15)	5 (3/63)	22 (15/67)	0 (0/65)
4	51 (23/45)	49 (22/45)	0 (0/1)	23 (9/40)	9 (4/43)	0 (0/45)
5	4 (9/211)	5 (10/205)	7 (2/30)	13 (24/192)	22 (41/190)	0.5 (1/210)
6	13 (7/54)	13 (7/54)	Not tested	4 (2/49)	13 (7/54)	0 (0/54)
7	42 (111/266)	47 (123/264)	1 (1/67)	3 (8/245)	16 (41/250)	0 (0/262)
8	18 (44/249)	21 (60/283)	0 (0/50)	3 (9/265)	10 (24/247)	0 (0/262)
9	17 (30/176)	14 (25/175)	9 (4/45)	10 (17/172)	12 (21/171)	0 (0/195)
10	1 (1/77)	1 (1/77)	67 (10/15)	7 (5/76)	13 (10/77)	0 (0/77)
11	2 (18/771)	5 (40/770)	44 (51/117)	3 (20/747)	5 (39/767)	0.1 (1/769)

Prevalence is shown as % (number samples positive/number samples tested).

**Figure 3:**
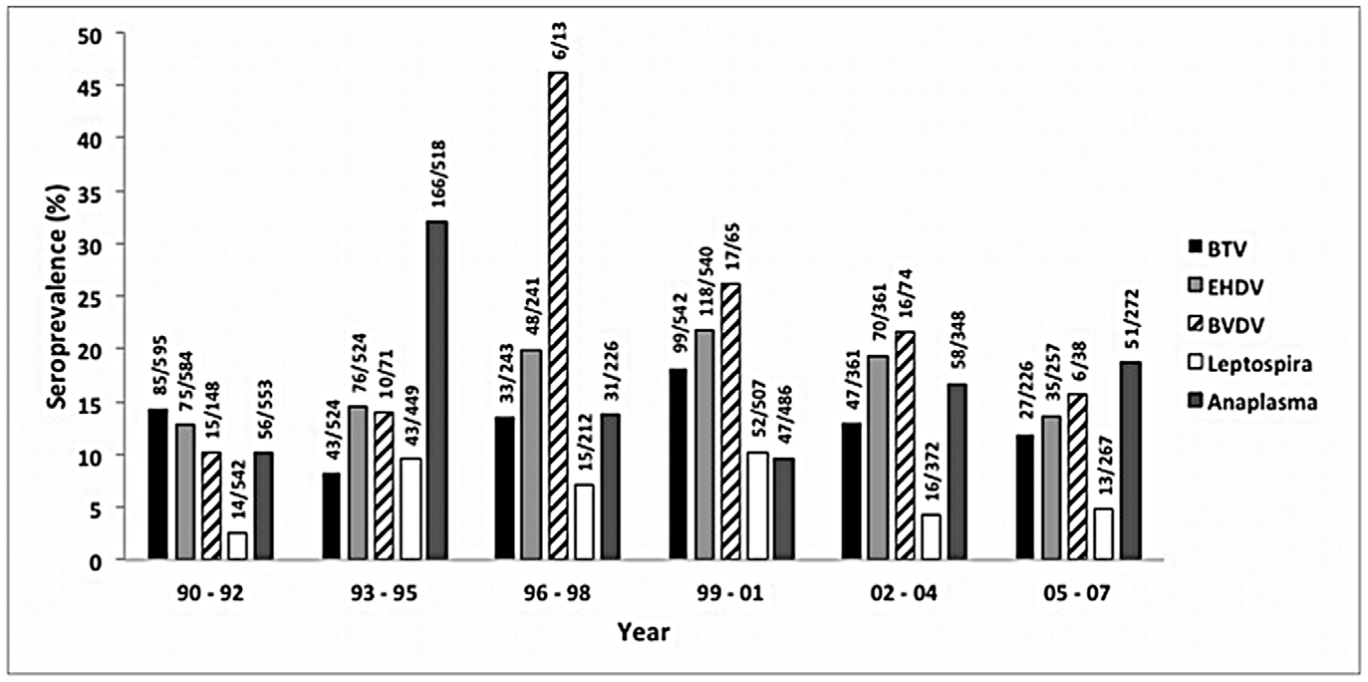
Seroprevalences by three year periods. Seroprevalence of bluetongue viruses (BTV), epizootic hemorrhagic disease viruses (EHDV), bovine viral diarrhea virus (BVDV), *Leptospira* spp. and *Anaplasma* spp. among California deer in three year intervals from 1990 to 2007.

Findings from the multivariate regression analyses indicated that age and area were significantly associated with exposure to BTV (Table 2). Deer >2 years of age had two times higher odds of being BTV seropositive than deer <2 years of age (*P*<0.001). BTV seroprevalence varied greatly by location and BTV seroprevalence was over 50% in area 4 in northern central California (23/45) and over 40% in area 7 in southern California (111/266) (Table 1, Fig. 2). Deer in these areas were over 44 (area 4) and 34 times (area 7) more likely to have been exposed than deer in area 11, which had a seroprevalence of 2%. The prevalence of BTV remained fairly consistent in time throughout the study period (Fig. 3). Over 80% of desert mule deer had evidence of previous exposure to BTV (44/54) and all of the positive samples in area 8 (44/249 tested) were from this subspecies. Because desert mule deer only occurred in area 8, data from desert mule deer were analyzed independently from other deer subspecies. Neither age nor sex was a significant predictor of BTV seropositive status among desert mule deer in area 8.

**Table 2:** Multivariate logistic regression model for exposure to bluetongue virus (BTV) and enzootic hemorrhagic disease virus (EHDV) among deer sampled at 11 deer assessment units (areas) in California between 1990 and 2007.

		BTV				EHDV		
	n	OR	95%CI	*P*-value	n	OR	95%CI	*P*-value
**Age**								
<2 years	460	1.0	ref	ref	459	1.0	ref	ref
>2 years	1830	2.1	1.4–3.2	<0.001	1811	2.0	1.49–2.7	<0.001
**Subspecies**								
Mule deer	1567	–	–	–	1594	1.0	ref	ref
Black-tailed	870	–	–	–	859	3.5	0.7–16.7	0.118
Desert mule deer	54	–	–	–	54	346.0	64.7–1849.7	<0.001
**Area**								
1	497	7.9	3.3–19.0	<0.001	489	2.1	0.4–11.6	0.403
2	80	3.6	1.2–10.4	0.019	80	0.7	0.1–3.8	0.648
3	65	5.9	1.3–27.0	0.022	65	1.7	0.3–8.9	0.536
4	45	44.8	7.3–276.5	<0.001	45	11.9	1.7–84.3	0.014
5	211	2.3	0.8–6.1	0.111	205	0.5	0.1–2.9	0.351
6	54	6.1	1.5–24.5	0.010	54	3.3	0.8–13.3	0.097
7	266	34.4	15.0–79.1	<0.001	264	23.4	12.4–44.1	<0.001
8	195	0	–	–	283	1.0	0.2–4.1	0.961
9	176	8.4	3.9–17.9	<0.001	175	2.8	1.5–5.5	0.002
10	77	0.6	0.1–3.0	0.491	77	0.2	0.1–1.7	0.130
11	771	1.0	ref	ref	770	1.0	ref	ref

0 = no samples were seropositive in this category.

Burro deer were not included in the model because of perfect correlation with area 8 and other deer subspecies were not significantly associated with BTV status.

ref = reference category.

Among deer exposed to EHDV, age, subspecies and area remained significant in the multivariate regression model (Table 2). As with BTV, deer >2 years of age were two times more likely to be EHDV seropositive than deer <2 years of age. Over 90% of desert mule deer sampled had evidence of previous exposure to EHDV (Table 1, Fig. 2) and desert mule deer were over 340 times more likely to be seropositive than other mule deer. The seroprevalences for EHDV varied geographically, but like BTV, seroprevalence was highest in area 4 in northern California and area 7 in southern California where over 45% of deer tested seropositive (Table 1). Deer in area 4 had 11 times higher odds of previous exposure and deer in area 7 had 23 times higher odds of previous exposure when compared to area 11 (Table 2).

Temporal patterns were also similar between EHDV and BTV (Fig. 3). The associations between the seroprevalences of BTV and EHDV and annual rainfall were explored in area 1, 5, 7, 8, 9 and 11 as these areas had more than 10 deer sampled in ≥7 years. No significant correlation was detected between average annual rainfall and seroprevalence of BTV or EHDV within these areas. However, for the overall larger dataset, there was a significant negative correlation between average annual rainfall within an area and the seroprevalence of both BTV (Spearman’s rho = −0.4220, P = 0.0002) and EHDV (Spearman’s rho = −0.2455, P = 0.0303). Results of the serologic tests for BTV and EHDV were highly correlated (*P*<0.001), which could indicate cross-reactivity between the tests, or similar risk factors for exposure to these pathogens, which share a common vector.

In the multivariate logistic regression analysis for BVDV, only area was significantly associated with BVDV exposure (Table 3). Generally, deer from eastern and northern California had the highest seroprevalence. Over 60% of deer tested in area 10 (10/15) had evidence of previous exposure to BVDV (Table 1, Fig. 2) and deer in this area had over 130 times higher odds of seropositive status than deer in area 7, which had a seroprevalence of 1%. However, sample size was low from area 10 and samples were only obtained in 1990–92 and 2002–04. The odds of seropositive status were also significantly higher in area 11 (Table 3). The overall prevalence may have been higher during the mid to late 1990’s but sampling effort was low during this time period (Fig. 3).

**Table 3:** Multivariate logistic regression model for exposure to bovine viral diarrhea virus (BVDV) and *Anaplasma* spp. among deer sampled at 11 deer assessment units (areas) in California between 1990 and 2007.

		BVDV				*Anaplasma* spp.		
Area	n	OR	95%CI	*P*-value	n	OR	95%CI	*P*-value
1	61	2.2	0.2–23.8	0.504	461	14.2	8.0–25.2	<0.001
2	8	0	–	–	76	2.2	0.7–6.7	0.169
3	15	0	–	–	67	5.4	0.9–31.3	0.061
4	1	0	–	–	43	1.9	0.3–10.7	0.460
5	30	4.7	0.4–59.4	0.230	190	5.1	2.8–9.4	<0.001
6	–	–	–	–	54	2.8	0.5–16.5	0.260
7	67	1.0	ref	ref	250	3.7	1.6–8.5	0.003
8	50	0	–	–	247	2.0	1.0–4.2	0.066
9	45	6.4	0.9–46.4	0.065	171	2.6	1.5–4.7	0.001
10	15	132.0	21.7–803.4	<0.001	77	2.8	0.4–18.3	0.286
11	117	51.0	7.0–371.7	<0.001	767	1.0	ref	ref

0 = no samples were seropositive in this category.

ref = reference category.

Only deer subspecies was significantly associated with previous exposure to *Leptospira interrogans*. Thirteen percent of black-tailed deer were seropositive (104/773) and this subspecies was over 4 times more likely to have been exposed to *Leptospira* than mule deer (*P-*value<0.001, Table 4, Fig. 2). Out of the 153 samples that tested positive, 78 (51%) had evidence of antibodies to *L. interrogans* serovar *pomona* (overall seroprevalence 3.3%), 42 (27.5%) had antibodies against serovar *grippotyphosa* (overall seroprevalence 1.8%) and 21 (13.7%) had antibodies against *L*. *interrogans* serovar *hardjo* (overall seroprevalence 0.9%). Less than 10% of the positive samples had antibodies against serovars *icterohaemorrhagiae* and *canicola* (overall seroprevalences less than 0.6%). The majority of samples that tested positive for antibodies to serovar *pomona* came from area 1 (43/78) and from black-tailed deer (60/78). Of the 42 samples positive for serovar *grippotyphosa*, most samples came from area 9 (13/42), area 1 (8/42) and area 5 (7/42), mainly among black-tailed deer (33/42). Serovar *hardjo* was most commonly (7/21) detected in area 5. When comparing seroprevalence between deer that were found dead or euthanized to deer that were live caught and apparently healthy, a significant difference was only found for *Leptospira* spp. with dead deer having 4.5 times higher odds of being seropositive than the live captured deer (95% CI: 1.9−10.9, *P*-value<0.001).

**Table 4:** Multivariate logistic regression model for exposure to *Leptospira* spp. among deer subspecies sampled between 1990 and 2007 in California.

		*Leptospira* spp.		
	n	OR	95%CI	*P*-value
**Deer Subspecies**				
Mule deer	1537	1.0	ref	ref
Black-tailed deer	773	4.7	2.7–8.4	<0.001
Desert mule deer	39	0	–	–

0 = no samples were seropositive in this category.

ref = reference category.

Exposure to *Anaplasma* spp. was most significantly related to location. *Anaplasma* seroprevalence was generally higher in coastal and northeastern California with 43% of deer in area 1 and 22% of deer in area 3 and 5 having evidence of previous exposure (Table 1, Fig. 2). Deer in area 1 had 14 times, and deer in area 3 and 5 had 5 times higher odds of exposure than deer in area 11 (Table 3).

Positive titers to *Brucella* were only detected in four samples; two deer from Alameda county in 1992 (female black-tailed deer >2 years of age), one deer from Mono county in 1994 (female mule deer <2 years of age), and one deer from Sacramento county in 1997 (male black-tailed deer >2 years of age). This very low 0.2% seroprevalence for *Brucella* is consistent with expected frequency of false positive tests results for a test with imperfect specificity.

## Discussion

Seroprevalence of BTV, EHDV, BVDV, *Leptospira* spp., and *Anaplasma* spp. among deer sampled in California in this study indicates that deer are widely exposed to pathogens that are of concern for livestock. In light of the recent emergence of pathogenic serotypes of BTV and EHDV and possible expansion of arthropod vector ranges, this baseline information is of value for monitoring the status of endemic diseases in deer and for evaluating the risk of disease transmission between wild ungulates and livestock. Location was the main determinant of seropositive status for most pathogens investigated here, while deer subspecies was the second most important predictor. Given that deer subspecies have fairly distinct, but overlapping geographic distributions, location clearly has a major influence on seropositive status for these pathogens. Geographic variation in the distribution of these pathogens highlights the value of large-scale monitoring to investigate the distribution of diseases potentially shared between livestock and wildlife.

We detected a high seroprevalence of both BTV and EHDV in southern and northern central California and found a much higher prevalence in desert mule deer for both BTV and EHDV than black-tailed deer and other mule deer. No previous information was available on the seroprevalence of BTV and EHDV in desert mule deer and our finding indicates that follow up studies investigating associated morbidity or mortality in this isolated population and identifying specific EHDV and BTV serotypes circulating in the Mohave and Sonoran desert are warranted. Desert mule deer are non-migratory and frequently inhabit riparian areas around the Colorado River and regional wildlife water guzzlers in southern California, which provide suitable habitat for the *Culicoides* spp. vector transmitting both BTV and EHDV. In comparison, seroprevalence to BTV and EHDV was low in eastern California which is inhabited by migratory mule deer that have distinct low-elevation winter ranges and high-elevation summer ranges less suitable for the *Culicoides* spp. vector. High seroprevalence rates were also detected in northern central California but previous information on the seroprevalence in deer from this region is not available for comparison. The prevalence of these two pathogens in area 7 (42% BTV, 47% EHDV), which is largely inhabited by Southern mule deer was consistent with seroprevalences detected by Chomel et al. (1994) who reported a 44% seroprevalence of BTV and 48% seroprevalence of EHDV in Southern mule deer from 1987–1991. The negative correlation between annual rainfall and BTV and EHDV seroprevalence detected in our study coincides with the geographical distribution of seropositive deer, with the highest seroprevalences found in southern California, which receives the lowest rainfall in the state. No significant correlations were found between precipitation levels and the seroprevalence of BTV and EHDV within specific areas, but scarce data may have influenced our ability to differentiate cyclical trends.

The average seroprevalence of 13.4% for BTV and 16.8% for EHDV observed throughout the study period is comparable to other studies from the American West, with 11% seroprevalence of BTV and 14% seroprevalence for EHDV in national parks in California, Colorado, Montana, Utah and Wyoming [43]. Higher rates of exposure among older age classes have been observed in previous serologic studies in California deer [36] and white-tailed deer in the southeastern United States [68]. Mortalities due to BTV and EHDV have not been reported in black-tailed or mule deer in California in the last two decades. However, epizootic hemorrhagic disease caused by adenovirus has been reported in California [69].

**Figure 4:**
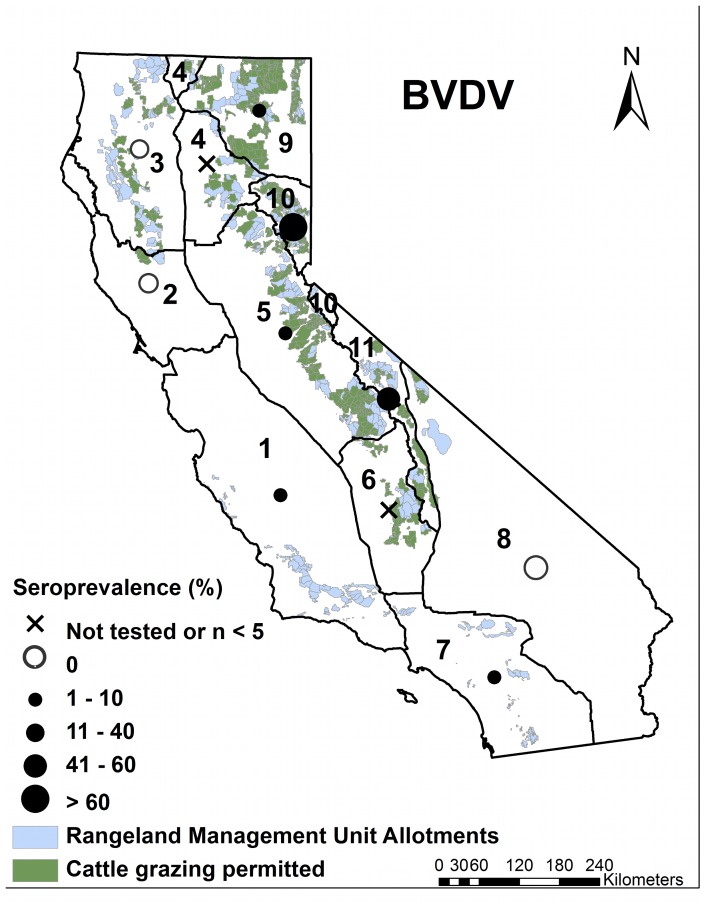
Seroprevalence of BVDV by deer assessment unit (area). Ranges of average bovine viral diarrhea virus (BVDV) seroprevalences among deer sampled between 1990 and 2007 in 11 deer assessment units (areas) in California. The distribution of public rangeland and cattle grazing land is also shown. Source: US Forest Service (http://www.fs.fed.us/r5/rsl/clearinghouse/gis-download.shtml).

The highest seroprevalence for BVDV was detected in eastern California (Fig. 2). In fact, the apparent increase in the overall seroprevalence of BVDV in 1997–1998 (Fig. 3) was caused by a larger proportion of samples coming from area 11 than other areas in these two years. Little information is available on the seroprevalence of BVDV in California deer except for one Yosemite National Park study that found four of seven (57%) deer sampled were seropositive [43]. A Minnesota study found a 20–22% seroprevalence of BVDV type 2 in adult white-tailed deer [70] whereas a study in Mexico found a seroprevalence of 63% [71]. In both studies, the seroprevalence was generally higher in areas with dense cattle populations. The high seroprevalence of BVDV in eastern California detected in our study overlapped with extensive federal grazing land (Bureau of Land Management, Forest Service) in this part of the state where direct contact between cattle and deer could occur (Fig. 4). Both white-tailed deer and mule deer are susceptible to infection with BVDV [20] and recent experimental studies have shown that white-tailed deer can produce persistently infected offspring when housed in close proximity to cattle [23] or with other persistently infected deer [22]. This suggests that BVDV can sustain itself in deer populations without contact with cattle. A very low prevalence of persistently infected animals have been found among free-ranging, hunter-killed, white-tailed deer in Alabama [72] and Colorado [73]. Some researchers have expressed concern that persistently infected wild ruminants could hamper BVDV eradication efforts in cattle [25], [26]. Currently, there is no government eradication program for BVDV, but the role wild ungulates play as a disease reservoir, or as a source of spillover or spillback of infections to and from cattle, will be critical information for any disease control effort.

The overall seroprevalence for *Leptospira* spp. found in our study (6.5%) was slightly higher than the 3% recorded in a 1979–1986 serosurvey in California [41]. Black-tailed deer had the highest odds for exposure and the geographic distribution of this subspecies corresponds with observations made by Behymer et al. (1989) indicating highest seroprevalences in the northwestern, north central and coastal areas of California. Northern California and the coastal ranges receive the highest amount of precipitation in the State [74] and these areas likely have more favorable environmental conditions for transmission of this pathogen. Serovars *pomona*, *grippotyphosa* and *hardjo* have previously been reported in white-tailed deer [27], [55], [71], [75] and the serovar *pomona* has been found to be associated with clinical leptospirosis in farmed deer [30], [76]. The serovar *hardjo* most commonly occurs in areas where deer have contact with cattle [71], and although the prevalence was low in our study, this serovar occurred most frequently in area 5 in central to eastern California where private and public grazing land could allow for contact between deer and cattle.

The low seroprevalence of antibodies against *Brucella* spp. in deer has been reported in previous studies that found a 0.06% seroprevalence among deer sampled between 1977 and 1989 [42] and no seropositive deer were detected in western national parks [43]. Several *Brucella* spp. have been documented in domestic species and *Brucella suis* occurs in feral swine in California [42]. The BAPA test used in this study has not been validated in deer, and pathogens known to cross-react with the test, such as *Yersinia enterocolitica* O:9, have been previously isolated from the environment and wild animals in California [77], [78].

For *Anaplasma* spp. the highest seroprevalences were detected in coastal and northeastern California which is consistent with the distribution of the main tick vector *Dermacentor* spp. [40]. These findings are in agreement with two previous California studies from 1979–1986 and 1987–1991 that documented a higher seroprevalence of *Anaplasma* spp. in the coastal areas [36], [41]. The overall seroprevalence during our entire study period (17.0%) was lower than the seroprevalence of 34% detected in 1979–1986 [41] and 56% detected in 1987–1991 [36]. This difference could be explained by lower sample size and a more limited sample distribution in the two previous studies, although our study was similarly limited by scarce data in many locations and time periods across this broad temporal and geographic scale.

This is the first report of high seroprevalence of BTV and EHDV among deer in northern California and the first larger serosurvey for BVDV in California deer. Seroprevalence of *Leptospira* spp., *Anaplasma* spp. and *Brucella* spp. among deer sampled extensively in this study was generally consistent with previous reports. Test validation and pathogen characterization will be important next steps for understanding disease transmission between deer and livestock, and longitudinal surveillance continues to be critical for understanding temporal patterns and monitoring the emergence of new serotypes that could be of concern to the livestock industry.
